# Integrated workflows for spiking neuronal network simulations

**DOI:** 10.3389/fninf.2013.00034

**Published:** 2013-12-10

**Authors:** Ján Antolík, Andrew P. Davison

**Affiliations:** Unité de Neurosciences, Information et Complexité, CNRS UPR 3293Gif-sur-Yvette, France

**Keywords:** Python, large-scale models, reproducibility, computational neuroscience, workflow, integration

## Abstract

The increasing availability of computational resources is enabling more detailed, realistic modeling in computational neuroscience, resulting in a shift toward more heterogeneous models of neuronal circuits, and employment of complex experimental protocols. This poses a challenge for existing tool chains, as the set of tools involved in a typical modeler's workflow is expanding concomitantly, with growing complexity in the metadata flowing between them. For many parts of the workflow, a range of tools is available; however, numerous areas lack dedicated tools, while integration of existing tools is limited. This forces modelers to either handle the workflow manually, leading to errors, or to write substantial amounts of code to automate parts of the workflow, in both cases reducing their productivity. To address these issues, we have developed Mozaik: a workflow system for spiking neuronal network simulations written in Python. Mozaik integrates model, experiment and stimulation specification, simulation execution, data storage, data analysis and visualization into a single automated workflow, ensuring that all relevant metadata are available to all workflow components. It is based on several existing tools, including PyNN, Neo, and Matplotlib. It offers a declarative way to specify models and recording configurations using hierarchically organized configuration files. Mozaik automatically records all data together with all relevant metadata about the experimental context, allowing automation of the analysis and visualization stages. Mozaik has a modular architecture, and the existing modules are designed to be extensible with minimal programming effort. Mozaik increases the productivity of running virtual experiments on highly structured neuronal networks by automating the entire experimental cycle, while increasing the reliability of modeling studies by relieving the user from manual handling of the flow of metadata between the individual workflow stages.

## 1. Introduction

One of the primary goals of computational neuroscience is to create models of neuronal structures and their function that as closely as possible adhere to the known anatomy of brain, while at the same time match as wide a range of experimental measurements as possible. In pursuit of this goal, supported by ever more plentiful computational resources, neuroscientists are building increasingly detailed, heterogeneous neuronal models and testing them under more and more elaborate experimental conditions. This process has accelerated in the last decade due to the increasing availability and capability of high-performance computing (HPC), enabling the simulation of neuronal structures at unprecedented levels of detail (Reimann et al., 2013), while expanding the simulations into multi-layer or even multi-areal contexts (Potjans and Diesmann, 2012; Nakagawa et al., 2013) and reproducing complex stimulation and recording experimental protocols (Shushruth et al., 2012).

**Figure 1:**
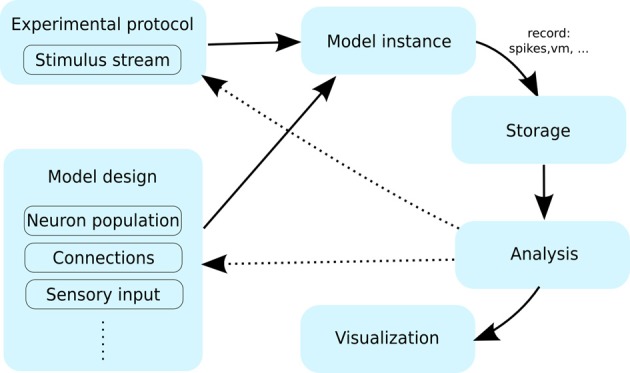
**The typical workflow of a neuronal simulation modeling project**. A user specifies the model using certain primitives such as neurons, the connections between them and the stimulation protocol. An instance of such a model is then simulated for a period of time, while being presented with stimuli specified by the experimental protocol. During the simulation a range of variables are recorded (e.g., spikes, membrane potential etc.); and typically stored for future processing. After the simulation is finished the data is; this data is then passed through the analysis and visualization protocols. At this point the user can inspect the results, which likely leads to modification of the model or experimental protocol (interrupted arrows), requiring another pass through the workflow cycle.

This rapid increase in the overall complexity of simulation workflows has only partially been accompanied by development of the various software tools that support them. The main development has been in the adaptation of neuronal network simulators to the HPC and supercomputer environments (Plesser et al., 2007), but the rest of the simulation workflow has been largely ignored. Such a focus on the underlying network simulation software was reasonable in the past, as it constituted most of the software stack required for a given project, and was by far the most computationally demanding part of it. However, due to the recent increase in the complexity of the models, stimulation/recording protocols and the subsequent analysis and visualization of the results, the lack of software support for the full simulation workflow (see Figure 1) drives users to write increasing amounts of code to meet the requirements of their projects. It is our belief that this lack of support for the full simulation workflow greatly reduces the productivity of the field (Wilson, 2006) and constitutes a major challenge for future development of brain simulation infrastructure.

In this paper we address this problem by proposing a new integrated environment for the simulation of spiking neuronal networks, which attempts to unify the various aspects of the workflow involved in simulating heterogeneous spiking neuronal networks in realistic experimental contexts and the subsequent processing of the resulting data, into a single automated workflow. Even though the current state of tools in the field is rather fragmented, there are a number of software packages that cover certain aspects of the workflow very well. Consequently our goal was to reuse as many of these existing tools as possible, focusing mainly on writing intermediate code to facilitate seamless communication between them, although we still had to cover several unaddressed aspects of the workflow by developing new tools.

The first question we faced in the process of designing Mozaik was the choice of programming environment. Conveniently, this choice was made rather easy by the recent proliferation of neuroscience tools implemented in Python. Python itself is an ideal language for integration projects (Scripps and Sanner, 1999), and together with the packages such as PyNN (Davison et al., 2009) and Neo (Davison et al., 2011) it provides an ecosystem perfectly fitted for our needs. PyNN is a simulator-independent specification of neuronal models, that includes (among others) back-ends for the major simulators used today including NEST (Gewaltig and Diesmann, 2007) and NEURON (Hines and Carnevale, 1997). Using this tool as the low-level model specification language makes Mozaik more independent of the simulation environment, yielding a more universal tool. The Neo package defines a common object model for storing a wide range of data typically recorded in neuroscience experiments, and allows for its flexible labeling with additional metadata. Furthermore, Neo also contains an extensive number of back-ends that allow for conversion of a number of data formats to and from the Neo data structures.

Mozaik has a modular architecture, and a great deal of emphasis has been put on designing a hierarchy of interfaces that allow for replacing or adding modules at the desired level of abstraction, reducing the amount of new code required for implementing new or changing existing features. Mozaik currently covers the following aspects of the workflow: experiment and stimulation specification, simulation execution, data storage, data analysis and visualization. Even though the major added value of Mozaik is the specification of application programming interfaces (APIs) for the various steps of the workflow, it also provides a ready to use implementation of all these APIs. While the APIs are written in as general a manner as possible, and should encompass a very wide range of spiking neuronal network simulation projects, the components implemented are currently focused on the visual modality. In this paper we provide a general description of the Mozaik design, accompanied with several illustrative usage examples, and explain the reasoning behind the various design choices. We do not, however, provide here a full user guide; this can be found at https://github.com/antolikjan/Mozaik.

## 2. Design goals

The motivation behind Mozaik is to increase the productivity of neuronal simulation workflows by means of automation and a reduction of the amount of boiler-plate code a user has to write. Most of the design principles discussed below are directly motivated by these two main goals.

### 2.1. Hierarchical abstraction

A common way of writing modular software is to partition the project into several (usually roughly equal) components, at roughly the same level of abstraction, and defining an independent API for each of them. This means that users can replace each of these components with their own implementation as long as they adhere to that component's API. The downside of such a “flat” strategy is that even small changes to the functionality of a given component generally require it to be completely rewritten (albeit using mainly copy-and-paste). The alternative strategy is to define a hierarchy of APIs at different levels of abstraction, which allows the user to pick the lowest point in the hierarchy that encompasses the functionality he intends to add or override, and hence minimize the amount of new code. In designing Mozaik we have employed the latter strategy.

### 2.2. Declarative configuration

There are two principal ways of specifying dynamical systems: imperative, where a user specifies the algorithm that will perform the desired actions; and declarative, where a user specifies what should be the outcome of the desired actions. Each has its advantages and drawbacks, with imperative specification being more flexible, and declarative more succinct, simpler and more human readable. In Mozaik we combined both approaches to gain both advantages where most appropriate. Generally Mozaik is a programmatic API, implying its imperative nature, which is inevitable given the scope of problems it addresses. However, we have pushed a declarative configuration functionality into all aspects of Mozaik, and paid attention to designing it to be as flexible as possible. In practice this means that when creating a new project most users will almost exclusively deal with the declarative configuration mechanisms and will write at most a few small, well isolated pieces of code (such as new analysis or visualization routines).

### 2.3. Balance between automation and flexibility

As declared in the Introduction the principal goal of Mozaik is to automate the workflow of neuronal network simulation projects. However, as with most automation projects, there is a compromise between the level of automation that can be achieved and the flexibility of the underlying framework in terms of the range of projects/configurations it supports. In Mozaik a good level of both automation and flexibility is achieved via the dual system of programmatic API and configuration front-end described above. If for a given project all required modules are already present in Mozaik, the project can simply be configured and the whole workflow will be executed without user intervention. On the other hand if the current set of Mozaik modules does not support some aspect of the project, a new module can be added, and the amount of required code should be minimized thanks to the hierarchical design of the API. Furthermore, special attention was paid to designing the analysis and visualization APIs (which are most likely to be extended by users) to automate the flow of metadata and configuration information, allowing the user to write a minimal amount of new code to express the desired functionality.

### 2.4. Explicit handling of metadata

One of the major problems Mozaik addresses is handling the flow of data between its various components. Even though it is usually clear what the main inputs and outputs of a given component are, it is very common that extra contextual information is needed at a given point of the workflow to correctly interpret the data, whether it is the units of a recorded variable or the stimulus that was presented. The most common way to deal with this contextuality is that each component in the framework specifies what context it requires (i.e., what units it assumes the voltage to be in). This approach is simple, but has several drawbacks: it prevents automation without an extra translation layer; it makes the component implicitly dependent on changes in components arbitrarily far away in the framework structure; it makes it hard to test the sanity of the input; and generally such implicit instead of explicit handling of information is more error prone.

For this reason in Mozaik we adopted the policy that as much contextual information as possible (in most cases all) is stored directly in the objects that are being passed around. This means not only that the level of automation in the Mozaik package can be high, but also that should a user need to add or modify any of the components he can be sure that all relevant metadata needed for processing within the component will be directly available in the given programmatic context, resulting in more localized code.

### 2.5. Emphasis on generality of input

The most common way of designing programming APIs is to specify a narrow focus for each component, which translates into code containing functions or classes with very specifically defined inputs and outputs. This approach makes it much easier to write code given the fewer input options, and the code can be exhaustively tested against the narrow input/output specification, resulting in fewer bugs. The disadvantage is that the resulting library is less flexible and leaves the user with writing more code to incorporate its functionality into a given programming context.

Given the integrative nature of Mozaik and its focus on minimization of boilerplate code, in several parts of the framework we have opted for the opposite design strategy. Particularly in the analysis and visualization modules, where flexibility is paramount, we have designed the API so that the input to analysis and visualization methods is very general (essentially it is any collection of recordings and analysis objects) and it is the role of the given method to “apply” itself to as broad a subset of this collection as possible, given its specification. This in turn allows us to have a unified system for filtering of such recording and analysis collections, which serves as a powerful tool for modifying the behavior of the analysis and visualization methods. Altogether this results in a more flexible system, allowing for the expression of complex analysis and visualization paradigms with minimal code. The disadvantage is that it makes the implementation of new analysis or visualization classes somewhat more complicated, thus shifting the balance between flexibility and ease of implementation toward the former.

### 2.6. Emphasis on full automation over interactivity

When designing a workflow one faces two basic options. Either it is fully automated, with a specification created in advance and then executed without user intervention, or the workflow is interactive, with the specification supplied on the fly. In Mozaik we chose the former option, as we believe it fits better the context of large-scale simulations, where the resource requirements make it rarely practical to run in an interactive fashion. One exception to this is the analysis and visualization modules, where users might in certain situations prefer interactive usage. While interactive use for these modules is in principle possible, the current design is focused on automation, making the process cumbersome. We hope to relieve this disadvantage in future with the addition of a dedicated GUI for these modules and/or integration of Mozaik with one of the analysis and visualization libraries that are taking shape in the field.

### 2.7. Other issues

In several cases (especially in the model specification module) we have faced design problems where flexibility of specification was in conflict with optimization of the code. In most of these cases we have valued flexibility over optimization.

Mozaik being an integrative workflow API, is by design a package that is supposed to grow over time as new model components, stimuli, experimental protocols, analysis and visualization methods are added. Furthermore, even the core API is likely to be changed in future as some parts of Mozaik will be taken over by dedicated tools. Therefore to ensure backwards compatibility and support future development the API is versioned, so that users can always be sure that their code is fully functional with respect to a well defined version of the library.

## 3. Architecture

To achieve the goal of automating all important parts of a typical large-scale neuronal simulation project workflow, Mozaik has to cover a wide range of heterogeneous functionality. Mozaik currently supports the following aspects of the workflow: experiment and stimulation specification, model specification, simulation execution, data storage, data analysis, visualization, and meta-workflow. To organize this complex structure and to support future changes we have structured Mozaik into nine sub-packages, each with its own API that is largely independent of the others:

1. **models**—specification of the high-level model API, and implementation of several basic models.
2. **sheets**—specification of the sheet API - Mozaik's basic model building block.
3. **connectors**—specification of API defining how neurons connect within and between sheets.
4. **stimuli**—stimulus specification API, unifies stimulus handling across the workflow.
5. **experiments**—API for implementation of experimental protocols.
6. **storage**—implementation of a data store, handling both raw recorded data and analysis results.
7. **analysis**—specification of the analysis API and implementation of a range of analysis methods.
8. **visualization**—specification of the visualization API and implementation of a range of plotting methods.
9. **meta-workflow**—code handling workflows involving multiple model instance execution (such as parameter searches).

It is our hope that several of these sub-packages will, in future, be separated out into stand-alone packages or replaced with third-party tools, as the software ecosystem in the field matures. In the remainder of this section we will first describe the high-level interaction between the components, and then discuss in more detail each of the sub-packages, focusing on the most important aspects of their design.

### 3.1. Control flow

The flow of control between the Mozaik components roughly follows the typical user workflow depicted in Figure 1. The workflow is launched from a short script which points Mozaik to a specification of a model, a list of experiments to be run with the model, and a list of analyses and visualizations a user wishes to perform on the recorded data. From this point, control is handed to Mozaik and the rest of the process is automated, resulting in a collection of files containing the raw recorded data, results of the analyses, including any intermediate steps, and possibly a set of figures saved in a specified format.

**Figure 2:**
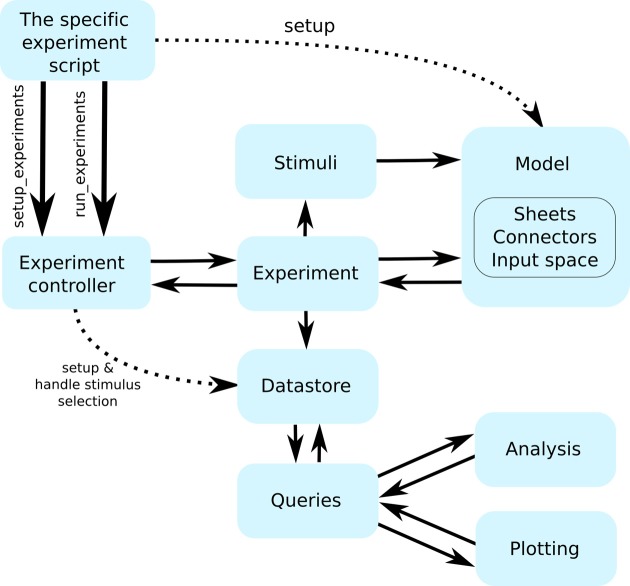
**The flow of control between the individual Mozaik components**. Continuous arrows indicate flow of control between components, while interrupted arrows indicate initialization of component instances. The workflow is initiated from a user script with a single command that hands control to the Mozaik control hub (the experiment controller), which in turn initializes the model and data store based on the parameter files specified on the command line and proceeds with executing the list of specified experiments one by one. Each experiment specifies a list of stimuli, which it sequentially presents to the model, executing the model simulation for each of them. At the end of each stimulus presentation the experiment module receives back the recorded data, which it passes into the data store module. Once all experiments are executed, a script specifying the list of analysis and visualization procedures to be run over the stored data is executed. The analysis and visualization routines directly communicate with the data store via a query interface in order to retrieve and manipulate the stored data.

To better understand the organization of Mozaik we will now give a brief account of how control flows between the components (see Figure 2). The user script hands control to the controller module, the control hub of Mozaik. The controller module creates a new data store, initializes the model instance (more on this in the following section), and proceeds to iterate over the experiment list. For each experiment it will ask for the list of stimuli that the experiment wishes to be presented to the model, and removes any that might have already been recorded in previously executed experiments, thus saving computing resources. After this it will hand over control to the experiment instance, together with the (possibly reduced) list of stimuli, which in turn will initiate the process of inserting the stimulus into the input space (see section 3.4) of the model and execute the simulation. In addition to specifying which stimuli will be presented to the model, an experiment can also add additional direct stimulation (for example current injection) of the model.

During simulation execution all raw data and relevant metadata are recorded and stored in the data store, based on the recording specifications that are part of the model's initialization (see section 3.6). At the point when all experiments have been executed, the data store holds all the recordings made during the experiments. At this point control flow returns to the controller module, which proceeds by handing the data store to the analysis and visualization methods, which will add additional analysis data to the data store and visualize them. Once this is done control returns again to the controller module which saves the final data store as the last step of the workflow. At this point the user can find all data including any possible figures in a pre-specified directory. Should he wish he can modify the list of analyses or visualizations to be done and run them on the saved data store without re-executing either the simulations or any previously performed analysis.

### 3.2. Parameters

In Mozaik we use two different systems for parameterizing components, each serving different roles: one for parameterizations of hierarchies of components using hierarchical configuration files, another for unified explicit parameterizations of data structures supporting a range of parameter specific operations. These parameterizations systems contribute significantly to the level of flexibility Mozaik offers, and play central roles in transmitting metadata between components—one of the major problems Mozaik addresses. The two systems are based around two external packages, (1) *parameters* (https://github.com/NeuralEnsemble/parameters) and (2) *param* (https://github.com/ioam/param), which Mozaik further extends.

The *parameters* package is used to parameterize hierarchies of objects for which the parameters are typically loaded from configuration files, and is thus used mainly in the components related to model specification. Each class in Mozaik that is parameterized via the *parameters* package has to specify a *required_parameters* attribute, a dictionary which holds the names and types of the required parameters. For such classes the presence of all the required parameters and the matching of their types with the supplied values is automatically checked. The type of a given parameter can also be *ParameterSet*, allowing for nesting of parameters, that can be then passed to other class instances created in the given class in a transparent fashion. Overall this allows configuring hierarchies of object in a transparent manner, separating the parameters from the code into human readable configuration files. Furthermore, the *parameters* package contains several useful operations with the parameter hierarchies, such as parameter references or parameter replacements, that greatly enhance the flexibility of working with such hierarchical specifications (see section 4.5).

The *param* package offers a way to explicitly declare attributes of a class as parameters, and associate additional information with them such as type, ranges or documentation strings. We extend it further in Mozaik to also declare units and periodicity. The advantage of the *param* package is that it allows for automation of processes dealing with the parameters due to their explicit declaration and the additional information associated with them, while retaining the standard Python attribute access in programatic context, resulting in more transparent and concise code. Specifically, in Mozaik there are several situations where we deal with large numbers of objects, each uniquely identified by a (potentially different) set of parameters. Often we want to refer to subsets of such collections of objects based on combinations of their parameter values. In Mozaik this happens when we deal with stimuli, with recordings and with analysis data structures. The *param* package facilitates common handling of these cases, and allows us to supply a single set of methods which provide powerful filtering of collections of such parameterized instances based on their parameter names and their values. As we will demonstrate in section 4 this system greatly enhances the flexibility of access to the records in the data store, allowing for simpler and more concise analysis and visualization code.

### 3.3. Model specification

Because the focus of Mozaik is large-scale heterogeneous neuronal simulations, it offers a higher-level abstraction for model specification than most other tools in the field. The four basic building blocks of a Mozaik models are:

1. **input space**—defines the geometry of sensory inputs.
2. **sensory input component**—defines how sensory input is translated into spiking of the peripheral neurons.
3. **sheet**—a population of neurons distributed in 2D space, typically corresponding to a certain type of neurons in a cortical layer.
4. **connectors**—a specification of a projection between two sheets of neurons.

The high-level input space API is very general, it basically only stipulates that stimuli are presented to the model at some discrete time steps, are embedded in some kind of vector space and can be added and removed from it. Currently only a single input space is implemented—visual modality, which corresponds to a 2D space with visual field coordinates. For this input space, a range of convenience methods is implemented, making adding, translating and scaling stimuli straightforward.

Each model that contains an input space (it is possible to specify models with no sensory inputs) must also contain a sensory input component that translates the stimulus in input space into spiking of neurons. Each such component declares one or several sheet components that will become the most peripheral neurons of the model, and which can be treated from the perspective of the rest of the model like any other sheet of neurons. The declared sheets of neurons do not have to be the physiologically most peripheral neurons (i.e., photo-receptors), for example the retina/LGN model that is shipped with Mozaik implements an abstract model of the whole pathway from photo-receptors to LGN relay neurons, and it declares only two sheets of neurons, corresponding to LGN ON and LGN OFF cell classes.

The sheet API is also very general, essentially it only declares a population of neurons that are distributed in 2D space, with respect to some coordinates. Multiple classes are derived from the base class that give additional meaning to the coordinates, such as classes that allow specification of cortical magnification factors with respect to visual field coordinates. This allows for more convenient specification of models, using more natural units such as densities, millimeters of cortical space etc., and it ensures automatic conversion of these parameters with respect to connectors or other sheets. Importantly the top level sheet class also contains extensive utility code allowing for specification of what to record, whether to apply some background stimulation, the possibility of adding additional metadata to the sheet that can be later utilized during analysis and plotting and shields from the user all the recording and data handling code.

Finally the high-level Mozaik connector component essentially corresponds to PyNN connectors, however, it allows for utilization of the extra information that is associated with the sheets to parameterize the connectivity, for example the position of the neurons. Indeed, to provide users with optimized connectors, we have created wrappers for all the existing PyNN connectors, which are thus available through the Mozaik connector API. However, on top of this high level API, that allows for writing for any potentially highly optimized connector functionality, we have built a flexible modular connector design. It is composed of two main components—the *ModularConnector* class, and the *ModularConnectorFunction*. The latter class defines a distribution of probabilities of connections between the pairs of neurons in the source and target sheets. The former serves as a container that combines multiple *ModularConnectorFunction* instances into the final probability distribution from which the connections are drawn. Importantly, it also defines how the connections will be drawn from the distribution. The specification of how the different *ModularConnectorFunction*s are combined is done via a Python expression that contains references to the declared *ModularConnectorFunction*s, making it very flexible to express different combinations of modular functions (see section 4.1 for an example).

### 3.4. Stimulus

The role of the stimulus component is two-fold, first is the actual generation of the stimulus temporal stream, and second is preservation of the stimulus identity throughout the Mozaik workflow. As we will show below, thanks to the *param* package these two roles are conveniently unified in a single class.

In Mozaik, each stimulus is a regularly sampled temporal stream of vectors of a certain dimensionality (2D in the case of visual stimuli). Each stimulus explicitly declares all the parameters (using the *param* package) that uniquely identify the stimulus. Each stimulus overloads a method that returns the vector stream corresponding to the stimulus for an arbitrarily long period of time. Importantly, Mozaik provides mechanisms that allow any class that is parameterized only via the *param* package parameters to be seamlessly converted into a so called ID object, which is essentially a shell of the original class holding only the parameters identities, their values, and the identity of the original class. This ID object can in turn be seamlessly converted back and forth into a string or the original stimulus instance.

During the recording, all data recorded during a presentation of a given stimulus are annotated with the corresponding stimulus ID object. This means that while only the identity of the stimulus and its parameters are stored with the recordings, at any point during the future processing the original stimulus can be easily recreated down the original vector stream used during the simulation. The identity of the stimulus is further maintained throughout the Mozaik analysis system, when it is passed into all generated analysis structures (see section 3.7 for more details). Last but not least the usage of the *param* package allows for utilization of the unified filtering functionality to perform searches on sets of records or analysis data structures annotated with the stimuli, based on the stimuli parameters, which allows for easier and more concise expression of analysis and visualization algorithms.

### 3.5. Experiment

As with most other Mozaik components, the high level experiments API is very simple and general. Each experiment is defined by the list of stimuli that it presents to the model and optionally a list of direct stimulations (i.e., current injection, etc.) that it administers during the duration of the experiment. Importantly, it is assumed that the stimuli are independent of each other—i.e., in real experiments they would be presented in randomized order and/or interleaved with presentation of blank stimuli. After presentation of each stimulus either a blank stimulus is presented or the simulation environment is reset (depending on the user's choice and availability of reset in the PyNN back-end simulator used), thus ensuring the independence of the responses on the order of the stimuli presentation. The length of the blank stimulus presentation is set by the user, and should reflect the dynamics of the model to ensure return of activity to background levels.

The independence of stimuli presentation is one of the central design decisions we took in Mozaik. It is based on the realization that the majority of stimulation protocols in neuroscience can be usefully subdivided into short presentations of independent stimuli, and that this allows for powerful conceptualization of the analysis and visualization code, leading to much more reusable code and a greater level of automation. Note that this does not prevent users from implementing experimental protocols where stimuli are not independent, but at the Mozaik level the user would have to concatenate all the dependent stimuli into one single longer Mozaik stimulus, thus losing some of the utility (but not all) of current Mozaik analysis and visualization components. It is possible that in future, a separate analysis package could be designed for analysis of such time dependent stimuli, but until now our usage of Mozaik did not indicate the necessity of this.

### 3.6. Storage and queries

The main role of the storage component is to accumulate recorded data annotated with all relevant metadata, as well as data structures produced during the subsequent analysis. The data store is represented by the class *DataStore* and is very simple: it essentially maintains two lists to which the recordings and analysis data-structures are added as they are produced. This is done automatically without user intervention. The storage component offers a number of methods to access the stored recordings based on the associated metadata. The storage of recordings is build around data-structures provided by the Neo package, while the analysis data structures (ADSs) will be discussed in section 3.7.

Importantly, the storage component also introduces the concept of views, implemented by the class *DataStoreView* (DSV), that represents an arbitrary subset of the data in the data store. The DataStoreView interface is identical to a “read-only” version of the *DataStore*, and thus allows for identical operations to be performed on a data store and its views, while preventing the replication of raw data in memory. The concept of views is utilized in query components, which allow a user to create new views of the data store, based on a given data store or data store view and various criteria, mostly related to associated metadata. Overall the storage components accompanied by the query system offer a powerful way to organize and filter data, which allows for highly flexible and reusable implementation of analysis and visualization components, as will be demonstrated below.

### 3.7. Analysis

A common way to implement analysis code is to specify methods that accept as input the data to be analyzed, in the form of data structures that are suitable for the given analysis. This allows for easier implementation of said analysis algorithm, but leaves a bigger burden to the future users as they will have to carefully study the input specification of each analysis method and often write a non-trivial amount of code that translates the data from their current form to the data structures required by the given analysis.

Given that the goal of Mozaik is automation and reduction of boiler-plate code, we have decided to shift the complexity from the usage of the analysis code to its implementation. Thus the high-level analysis API is extremely general—it essentially specifies that the input of each analysis is a *DataStoreView* instance. It is the role of the given analysis class to filter out the widest range of data from the data store view to which it can be applied. The results of analyses are ADSs that are stored back into the data store, to become potential future inputs to subsequent analysis or visualization steps.

ADSs are derived from the high-level abstract class *AnalysisDataStructure*. Similarly to the stimulus API, analysis data structures have to declare all their parameters using the *param* package; the only attributes they declare should be the ones that hold the “raw” data. This allows the query system to filter collections of ADSs based on any of their parameters. The *AnalysisDataStructure* class from which all ADSs are derived declares several parameters that are thus common to all ADSs:

1. **identifier**—which essentially corresponds to the given ADS class name.
2. **analysis_algorithm**—set to the algorithm that produces the specific ADSs instance.
3. **sheet_name**—sheet with which this ADS is associated.
4. **neuron**—neuron with which this ADS is associated.
5. **stimulus_id**—stimulus with which this ADS is associated.

The latter three parameters can also be set to None. For example if *neuron* is set to None it means that the ADS is not associated with a single neuron (i.e., when it holds population average results). Users can add new ADSs as required; however, they are encouraged to re-use existing ADSs whenever possible, as this allows for potentially more of the already implemented analysis and visualization methods to be applicable to the given results. So far our experience was that a wide range of analysis results can be expressed with very few different ADSs.

The parameters in the ADSs function as metadata that identify the content of the ADS. When specifying a new ADS it is important to think of all parameters that might be required to identify the results in the data store. In the same way, whenever a new analysis method is implemented, the developer has to make sure to use ADSs that are suitable for holding the results of the said analysis and set all parameters that are required to identify the results in the data store. Altogether this makes the process of writing new analysis methods somewhat more involved; however, as we will demonstrate in the following section, it leads to a very flexible and reusable analysis system, requiring a minimal amount of code from the end user.

### 3.8. Visualization

The philosophy of the visualization package is similar to that of the analysis sub-package. The input of each visualization method is a DSV. The role of each visualization method is to extract as much data as possible from the provided DSV and display as much information as possible with respect to its visualization role. It is thus up to the user to filter the data store before passing it to the plotting function to select the information that is plotted.

Beyond the flexibility of input, the other major design goals were to support hierarchical definition of figures, and separation of the “appearance” aspects of plotting from the rest of the visualization functionality. To achieve this the visualization package is divided into two separate APIs, represented by two high-level classes *Plotting* and *SimplePlot*, the former representing the high-level hierarchical functionality, while the latter represents low-level plotting.

The implementation of the *Plotting* API is based on matplotlib (Hunter, 2007) and its *GridSpec* objects. The *Plotting* API is mainly responsible for hierarchical organization of figures with multiple plots, any mechanisms that require consideration of several plots at the same time, and the translation of the data from the general format provided by DSVs, to any specific format that the low-level *SimplePlot* classes require. In general the *Plotting* instances do not do any plotting of axes themselves, but instead call the *SimplePlot* instances to do the actual drawing. Each class that was derived from *Plotting* derived from plotting can itself contain several other *Plotting* or *SimplePlot* references. Essentially each figure in Mozaik is a tree, whose inner nodes are *Plotting* instances and leaves are *SimplePlot* instances. Each *Plotting* instance contains a GridSpec object, that defines how the figure space at that level of hierarchy is separated between the children components. This allows for very concise and flexible constructions of complex figures with multiple plots.

The *SimplePlot* API represent low-level plotting. Each *SimplePlot* represents only a single matplotlib axis that is drawn into the region defined by the GridSpec instance that is passed into it. The high-level *SimplePlot* API offers a range of mechanisms to control the detailed look of the plot. When defining a new *SimplePlot* class the user is encouraged to use, as much as possible, the decorating mechanisms provided by the high-level *SimplePlot* API. This ensures a unified look for all Mozaik figures, while maintaining a good appearance for new plots with a minimal amount of code.

## 4. Example usage

**Figure 3:**
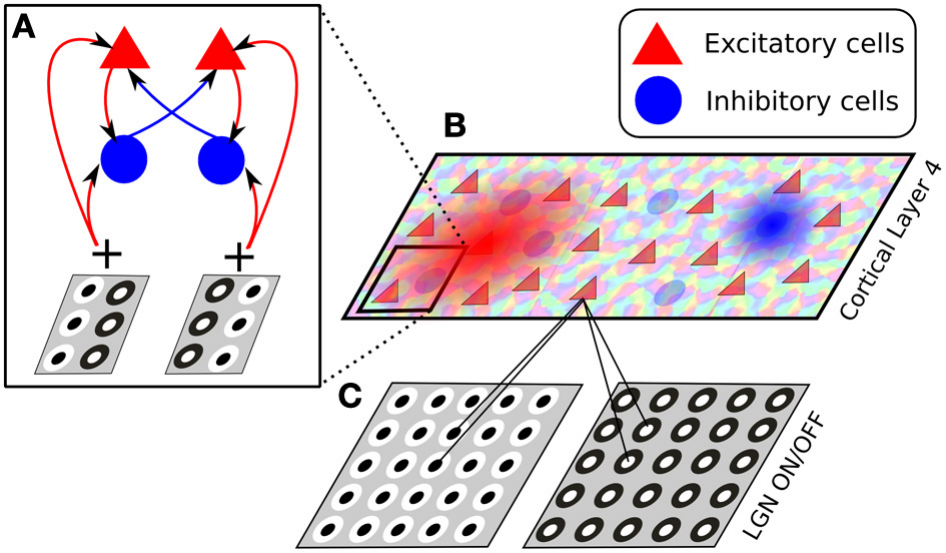
**The architecture of the model presented in section 4**. The model consists of two thalamic layers, corresponding to ON and OFF cell types **(C)**, and a single cortical layer of excitatory and inhibitory cells, whose afferent receptive fields are generated drawn from a Gabor-shaped probabilistic distribution, whose parameters are based on an orientation map **(B)**. The connectivity between cortical neurons is set to have push-pull connectivity, i.e., excitatory neurons selective to a certain orientation and phase connect with a higher likelihood to other excitatory or inhibitory neurons preferring the same orientation and phase, while inhibitory neurons are more likely to connect to excitatory and inhibitory cells preferring the same orientation but opposite phase **(A)**.

We will now walk through an example Mozaik project to demonstrate how the design principles discussed in the previous sections translate into a real-world scenario. The following project creates a simple model of visual cortex (see Figure 3), probes it with a standard stimulation protocol using sinusoidal gratings, and applies several analysis and visualization algorithms commonly performed in visual cortex.

### 4.1. Model specification

As discussed in section 2.2, if all required components are already implemented in Mozaik, a new model can be declared almost entirely via configuration files. Mozaik expects as input a single hierarchical configuration structure. However, the *parameters* package allows us to conveniently split this hierarchy into multiple files for better organization and reuse. Below we show the top level configuration file, which specifies several high-level parameters and also contains references to files containing the configurations of individual sheets of neurons (in this case representing cortical layers):


{   'input_space_type': 'mozaik.space.VisualSpace',
    'input_space': {
        'update_interval': 7.0, # ms
        'background_luminance': 50.0 }, # cd/m^2
    'retina_lgn': url("param/SpatioTemporalFilter
                       RetinaLGN_defaults"),
    'l4_cortex_exc': url("param/l4_cortex_exc"),
    'l4_cortex_inh': url("param/l4_cortex_inh"),
    'visual_field': {
        'centre': (0.0, 0.0), # degrees (x, y)
        'size': (6.8, 6.8) }, # degrees
                                (width, height)
    'results_dir': ",
    'name': 'V1 Model',
    'reset': False,
    'null_stimulus_period': 140.0 }

Note the *url* keyword that allows us to refer to another file containing the corresponding sub-tree of the hierarchical configuration. Let us now have a look at configuration of the excitatory sheet. As it is rather long we will look at it in parts. First are the parameters configuring the excitatory sheet of neurons itself:


'component': 'mozaik.framework.sheets.
              CorticalUniformSheet',
'params': {
    'name': 'V1_Exc_L4',
    'sx': 5000.0,
    'sy': 5000.0,
    'density': 64.0,
    'mpi_safe': True,
    'magnification_factor': 5000.0,
    'cell': {
        'model': 'IF_cond_exp',
        'params': {
            'v_thresh': −57.0,
            'v_rest': −70.0,
            'cm': 0.29,
            'tau_m': 10.0,
            ... },
        'initial_values': {
            'v': PyNNDistribution(name='uniform',
                            params=(−60, −50))}},
    'background_noise': {
        'exc_firing_rate': 2000.0,
        'exc_weight': 0.00145,
        'inh_firing_rate': 2000.0,
        'inh_weight': 0.00030 },
    'recorders': url("param/l4_exc_rec") }

Here the parameter *component* contains the reference to the sheet class we want to use, in this case a cortical sheet with spatially uniformly distributed neurons. The parameter *params* then contains a hierarchy of parameters that will be passed to the sheet class. At the top we see several parameters directly configuring the sheet. Next the *cell* parameter specifies the PyNN neuron model to be used, its parameters, and initial conditions. At the bottom we see a parameter *recorders* which holds configurations for a set of recorder objects specifying which variables in which neurons will be recorded during the simulation. For example, the following configuration will tell Mozaik to record spikes from 1000 randomly selected neurons, and to record the membrane potential and synaptic conductances from another 20:


"Spikes" : {
    'component': 'mozaik.sheets.population_selector.
                                         RCRandomN',
    'variables': ("spikes",),
    'params':  {'num_of_cells': 1000} },
"DetailedRecordings": {
    'component': 'mozaik.sheets.population_selector.
                                         RCRandomN',
    'variables': ("spikes","v","gsyn_exc","gsyn_inh"),
    'params':  {'num_of_cells': 20} }

Following the sheet parameters, the configuration file also contains the specification of the connectors. First let us look at the lateral connections (see Figure 3B):


'L4ExcL4ExcConnection': {
    'target_synapses': 'excitatory',
    'short_term_plasticity': {
        'u': 0.5,
        'tau_rec': 1100.0,
        'tau_psc': 1.5,
        'tau_fac': 50.0 },
    'weight_functions': {
        'f1': {
            'component': 'mozaik.connectors.modular_
        connector_functions.V1PushPullArborization',
            'params': {
                'or_sigma': 0.26,
                'phase_sigma': 0.52,
                'target_synapses': 'excitatory' }},
        'f2': {
            'component': 'mozaik.connectors.modular_
    connector_functions.HyperbolicConnectorFunction',
            'params': {
                'theta': 207.76,
                'alpha': 0.013944 }}},
    'delay_functions': {},
    'weight_expression': 'f1*f2',
    'delay_expression': '2',
    'base_weight': 0.108,
    'num_samples': 72 },
'L4ExcL4InhConnection':
         ref('l4_cortex_exc.L4ExcL4ExcConnection'),

Here we specify two connectors corresponding to the connections from neurons in the excitatory sheet to other neurons in the excitatory and inhibitory sheets (the parameters *L4ExcL4ExcConnection* and *L4ExcL4InhConnection*). Each connector allows the user to specify the short term plasticity model to be used for the synapses. The *L4ExcL4ExcConnection* connector uses the Mozaik modular connector specification, which allows the user to specify a number of connector functions to determine the weights and delays of connections (the *weight_functions* and *delay_functions* parameters). The connector functions are then combined in the *weight_expression* and *delay_expression* parameters to determine the distributions of delays and weights.

Here we define two connector functions for weights, one specifying that neurons are connected in a push–pull manner with respect to the orientation of their receptive field (see Figure 3A), while the second connector function specifies that the connection probability between neurons falls off with distance following a hyperbolic function (see Figure 3B). By specifying the *weight_expression* as a multiplication between the two connector functions we effectively say that the probability of the connections of neurons will be falling of with distance, while with respect to orientation similarly-oriented neurons will be more probably connected. In this connector we want to have all connection delays be a constant of 2 ms, which we can simply express by setting the *delay_expression* parameter to 2, without specifying any connector functions for delays.

The specification of the *L4ExcL4InhConnection* connector parameter demonstrates another feature of the configuration system: references. In this example we want the connections from excitatory to inhibitory neurons to follow the same rules. Therefore instead of repeating the whole configuration structure specified for the *L4ExcL4InhConnection* we simply give a reference to it.

The following listing shows the specification of the third afferent projection in the model from thalamic to cortical neurons, which is based on externally supplied orientation map (see Figure 3B):


'AfferentConnection': {
    'aspect_ratio':
                  UniformDist(min=0.57, max=0.57),
    'size':
                  UniformDist(min=0.46, max=0.46),
    'orientation_preference':
      UniformDist(min=0.0, max=3.141592653589793),
    'phase':
      UniformDist(min=0.0, max=6.283185307179586),
    'frequency':
                    UniformDist(min=0.8, max=0.8),
    'or_map_location' : './or_map',
    ...
    'specific_arborization': {
        'weight_factor': ref('l4_cortex_exc.L4ExcL
                   4ExcConnection.base_weight')*2,
        'num_samples': 94,
        'target_synapses': 'excitatory',
        'short_term_plasticity': { ... }}}

Apart from supporting more concise model specifications, the references can also be combined in simple Python expressions as demonstrated in the parameter *weight_factor* of the *AfferentConnection* connector specification. Here we say that the weights of this connector should be twice as strong as those of the lateral connections. This provides a very flexible way of defining the most common relationships between model parameters and, as will be demonstrated in section 4.5, greatly simplifies the specification of parameter search protocols.

To finish specification of the model, the user currently has to still write a short Python class. For the model discussed here the model class would look as follows:


class PushPullCCModel(Model):

    required_parameters = ParameterSet({
        'l4_cortex_exc': ParameterSet,
        'l4_cortex_inh': ParameterSet,
        'retina_lgn': ParameterSet ,
        'visual_field': ParameterSet
    })

  def __init__(self, sim, num_threads, parameters):
    Model.__init__(self, sim, num_threads,parameters)
    # Load components
    CortexExcL4 = load_component(self.parameters.
                            l4_cortex_exc.component)
    CortexInhL4 = load_component(self.parameters.
                            l4_cortex_inh.component)
    RetinaLGN = load_component(self.parameters.
                               retina_lgn.component)

    # Build and instrument the network
    self.visual_field = VisualRegion(location_x=self.
         parameters.visual_field.centre[0],
         location_y=self.parameters.visual_field.
                    centre[1],
         size_x=self.parameters.visual_field.size[0],
         size_y=self.parameters.visual_field.size[1])

    self.input_layer = RetinaLGN(self,
                   self.parameters.retina_lgn.params)
    cortex_exc_l4 = CortexExcL4(self,
                self.parameters.l4_cortex_exc.params)
    cortex_inh_l4 = CortexInhL4(self,
                self.parameters.l4_cortex_inh.params)

    # initialize each of the projections
    GaborConnector(self, self.input_layer.sheets
          ['X_ON'], self.input_layer.sheets['X_OFF'],
          cortex_exc_l4, self.parameters.l4_cortex_
                            exc.AfferentConnection,
          'V1AffConnection').connect()
    GaborConnector(self, self.input_layer.sheets
          ['X_ON'], self.input_layer.sheets['X_OFF'],
          cortex_inh_l4, self.parameters.l4_cortex
                            _inh.AfferentConnection,
          'V1AffInhConnection').connect()
    ModularSamplingProbabilisticConnector
          (self, 'V1L4ExcL4ExcConnection',
           cortex_exc_l4, cortex_exc_l4, self.para
           meters.l4_cortex_exc.L4ExcL4ExcConnection
           ).connect()
    ...

Essentially all this code does is to initialize the individual sheets and then use them to initialize the individual connectors. It should in principle be possible to automate the above code, and thus we plan that in future releases of Mozaik the user will be able to skip this configuration step altogether.

### 4.2. Experiments and execution

Each mozaik experiment specifies a list of stimuli that will be presented to the model, and optionally what direct stimulation (such as current injections) happens throughout the duration of the experiment. To tell Mozaik what experiments to execute over the model the user has to create a function that accepts the model as a parameter and returns a list of initialized experiments like this:


def create_experiments(model):
 return [
  MeasureSpontaneousActivity(model, duration=147*7),
  MeasureOrientationTuningFullfield
    (model, num_orientations=2, spatial_frequency=0.8,
     temporal_frequency=2, grating_duration=147*7,
     contrasts=[10, 20, 30, 40, 50, 60, 70,
                80, 90, 100],
     num_trials=10),
  MeasureNaturalImagesWithEyeMovement(model,
             stimulus_duration=147*7, num_trials=15) ]

Here we tell Mozaik to perform three experiments: one during which there is no stimulation (which effectively means we measure spontaneous activity); one where we perform a full-field orientation tuning protocol; and one in which we show a natural image movie with simulated eye movements.

At this point the whole workflow is configured and we are ready to execute it with a single command, to which the model class and a method returning a list of experiments is passed:


data_store, model = run_workflow('PushPullModel',
               PushPullModel, create_experiments)

Running the above command hands control to Mozaik, which will proceed to execute each of the specified experiments while recording all the specified variables. All recorded data will be annotated with relevant metadata and stored in a central data-store that is handed back to the user for subsequent analysis and visualization. Mozaik will report the progress of the stimulation, by logging the number of already executed experiments and presented stimuli at the end of each stimulus presentation. This log is by default routed both to the standard output and a file.

### 4.3. Data storage and manipulation

Once the Mozaik workflow is executed all data are stored in the central data store. The Mozaik query system allows the user to express operations over the data, with respect to the metadata with which they are annotated, in a simple manner. For example, the following query returns a DSV with data (recordings or analysis data structures) associated only with sheet *V1_Exc_L4*:


param_filter_query(data_store,
                   sheet_name=['V1_Exc_L4'])

while the following code filters data structures that declare a parameter named *value_name* with value *AfferentOrientation* and that belong to any of the listed sheets:


param_filter_query
        (data_store, sheet_name=['V1_Exc_L4',
         'V1_Inh_L4', 'V1_Exc_L2/3', 'V1_Inh_L2/3'],
         value_name='AfferentOrientation')

Finally, the following query filters all recordings or analysis data structures associated with any sheet and recorded during presentation of stimulus *FullfieldSinusoidalGrating* of horizontal (0°) orientation:


param_filter_query(data_store,
                 st_name='FullfieldSinusoidalGrating',
                 st_orientation=0)

Here we demonstrated simple filtering queries, but Mozaik offers a number of other query methods providing more complex data manipulation, such as collation of data with respect to selected parameters. These significantly simplify the expression of a range of visualization and analysis algorithms.

### 4.4. Analysis and visualization

Now that we have explained how to access and filter data in data-store we can proceed to explain the analysis and visualization system. To remind the reader, the input to each analysis or visualization method is a DSV, they should always try to apply themselves to the largest possible subset of DSVs, and the analysis methods produce ADSs that are put back into the data store to become inputs for subsequent analysis and visualization. This, combined with rich annotation of recorded data, ADSs with metadata and the query system, allows for a powerful, flexible, and unified way of modifying the behavior of the analysis and visualization process.

**Figure 4:**
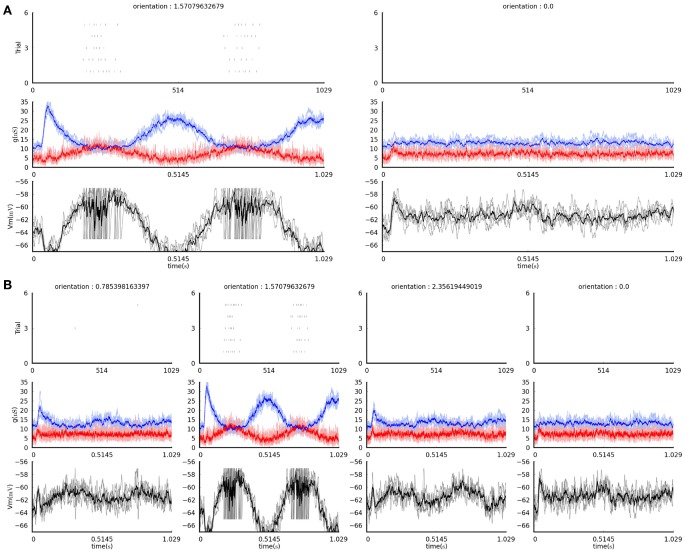
**The figures produced by the example visualization commands in section 4.4 (see text)**. **(A)** The result of the first command, plotting only the data acquired during presentation of vertically and horizontally-oriented gratings. **(B)** The result of the second command, plotting data acquired during presentation of all grating orientations (four, in this case). In both plots, each column shows recordings in response to a grating of the orientation indicated at the top. The top panels show the spike rasters for the five times each stimulus was presented. The middle panel shows the excitatory (red) and inhibitory (blue) conductances recorded during the stimulus presentation; the pale lines show single trial traces while the thick saturated lines show the trial average trace. The bottom panels show the membrane potential traces recorded during the stimulus presentation; the gray lines showing the single trial traces while the black line shows the trial averaged trace.

Let us now have a look at several simple examples demonstrating this functionality. The following code visualizes the raw data recorded during presentation of a *FullfieldSinusoidalGrating* stimulus at horizontal and vertical orientations and 100% contrast (Figure 4A):


dsv = param_filter_query(data_store, st_orientation
       =[0, pi/2],
       st_name=['FullfieldDriftingSinusoidalGrating'],
       st_contrast=100)
OverviewPlot(dsv, ParameterSet({'sheet_name':
       'V1_Exc_L4', 'neuron': l4_exc,
       'sheet_activity': {}}))

while this code will do the same, but will display the data for any orientation of the grating stimulus that was presented and recorded (Figure 4B):


dsv = param_filter_query(data_store,
    st_name=['FullfieldDriftingSinusoidalGrating'],
    st_contrast=100)
OverviewPlot(dsv, ParameterSet({'sheet_name':
    'V1_Exc_L4', 'neuron': l4_exc,
    'sheet_activity': {}}))

In the above two examples the first line restricts the data store to the set of responses we wish to visualize, while the second line is a basic visualization method that shows the raw responses to each stimulus recorded in the data store.

We can use the same system to control the analysis scope. For example, this is how to compute trial-averaged firing rates to all the presented stimuli:


TrialAveragedFiringRate(data_store).analyse()

while this is how one would compute trial-averaged firing rates only for neurons in a sheet named *V1_Exc_L4* and only due to a *FullfieldDriftingSinusoidalGrating* stimulation.


dsv = param_filter_query(data_store,
    st_name=['FullfieldDriftingSinusoidalGrating'],
    sheet_name='V1_Exc_L4')
TrialAveragedFiringRate(data_store).analyse()

Behind the scenes, this code creates a number of ADSs—one per stimulus—holding the average firing rates of individual neurons, and adds them back into the data store. These analysis data structures are annotated with metadata such as the identity of the stimulus with which they are associated, allowing for their identification using the query system.

**Figure 5:**
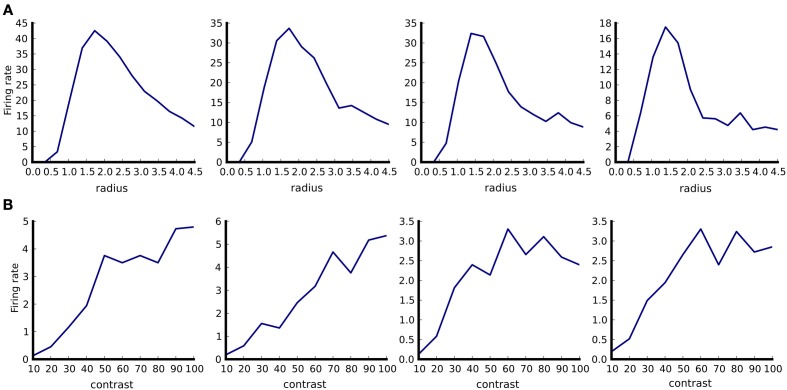
**Example of tuning curve plotting**. Responses of four neurons in responses to optimally oriented drifting sine-gratings disks confined to an aperture of varying diameter **(A)**. Responses of the same four neurons as in **(A)** to optimally oriented full-field drifting sine-gratings of varying contrast **(B)**. Firing rates were calculated as spike counts during the one second stimulus presentation and averaged over ten trials. The aperture diameter is in degrees of visual field.

After populating the data store with ADSs holding a single value per stimulus and neuron (specifically the trial-averaged firing rate), we can for example plot the tuning curves that span the parameter space of the presented stimuli. The following code would plot the size tuning curves of neurons in *V1_Exc_L4*, based on the trial averaged firing rate responses due to the *DriftingSinusoidalGratingDisk* stimuli:


dsv = param_filter_query(data_store,
       st_name='DriftingSinusoidalGratingDisk',
       analysis_algorithm=['TrialAveragedFiringRate'],
       contrast=100, sheet_name="V1_Exc_L4")
PlotTuningCurve(dsv,
       ParameterSet({'parameter_name': 'size',
       'neurons': list_of_4_neurons})).plot()

(see Figure 5A) while the following code would plot contrast response curves (Figure 5B):


dsv = param_filter_query(data_store,
       st_name='DriftingSinusoidalGratingDisk',
       analysis_algorithm=['TrialAveragedFiringRate'],
       size=1.5, sheet_name="V1_Exc_L4")
PlotTuningCurve(dsv,
                ParameterSet({'parameter_name':
                'contrast', 'neurons': list_of_4_
                neurons})).plot()

Note that the *PlotTuningCurve* visualization function is agnostic to how the values it is plotting have been computed and through which parameter we want to plot the tuning curve. It simply expects a set of ADSs in the datastore view (specifically ADSs of type *PerNeuronValue*, which hold a single scalar value per neuron) which are associated with the same kind of stimulus but with varying values of its parameters. By telling it through which parameter we want to plot the tuning curve it has all the information required to perform the task. Furthermore, because the *PerNeuronValue* ADS holds other useful metadata, such as the name of the value that it stores, and the units in which it was measured, the *PlotTuningCurve* can use these to populate the plot with all this contextual information. This demonstrates how our system allows for specification of very general operations that can be used in a broad range of contexts, making them much more reusable.

### 4.5. Meta-workflow

The meta-workflow sub-package encapsulates code related to running workflows that require execution of multiple model instances with different parameterizations and the processing and visualization of the data produced by them. Currently, the commonly used grid parameter search is implemented. Thanks to the flexible parameterizations system discussed and demonstrated in previous sections, execution of a systematic parameter search of a model such as the one described above is possible with a single line of code:


CombinationParameterSearch(
  SlurmSequentialBackend(num_threads=4, num_mpi=8),
   { 'l4_cortex_exc.L4ExcL4ExcConnection.weights':
      [0.1, 0.2, 0.3, 0.4, 0.5],
     'l4_cortex_inh.L4InhL4ExcConnection.weights':
      [0.2, 0.4, 0.6, 0.8, 1.0] }).run_parameter_
      search()

Here the first parameter specifies which job scheduling back-end to use (here the back-end for Slurm (Jette et al., 2002)), and the second parameter is a dictionary with parameter paths as keys and lists as values. Thanks to the possibility of using references in parameter files, it is very easy to express relationships between parameters such as keeping a balance between excitatory and inhibitory weights constant while varying its magnitude, etc. The above code will schedule 25 simulations with all combinations of values of the two selected parameters. All the results will be stored in a master directory together with extra information about the parameter search that can be used for further processing of the data. Data from each simulation is stored in a separate sub-directory, together with any figures produced during the given simulation run.

The most common way of analyzing and visualizing parameter search data is to implement a measure producing single value per model instance (e.g., mean firing rate of the model neurons) and than show it as a function of the varied parameters. For such situations Mozaik offers a visualization method that automatically collates the produced data and displays them:


single_value_visualization("PushPullModel",
       "./PushPullModelParameterSearchRun1",
       ParamFilterQuery(ParameterSet({'params':
       ParameterSet({'sheet_name': 'V1_Exc_L4'})})),
       filename='ExcitatoryPopulation.png')

**Figure 6:**
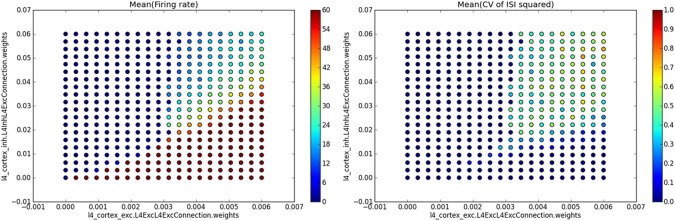
**Example of automatic plotting of parameter search results**. Method *single_value_visualization* plots the values from *SingleValue* ADSs as a function of the parameters that were varied (see the axis labels in the plots which specify the full path of the parameter in the parameter hierarchy), creating one subplot for each value of parameter *value_name* (see titles of the subplots). In this specific example we ran a simulation of the Vogels and Abbott (2005) model, while varying the strength of excitatory and inhibitory synapses in the network. For each combination of values of these two parameters the network's mean firing rate and mean square of coefficient of variation of inter-spike intervals (CV of ISI) were calculated, thus providing a single value measure for each network parameterization. The plot on the left shows the mean firing rate as a function of the varied parameter values, while the plot on the right shows the mean square of CV of ISI measure.

Here the first two parameters identify the model that was run and the master directory in which the parameter search data were stored, the third parameter is a query instance that will be applied to filter out data from data-stores produced during each of the individual simulation runs, and the last parameter specifies the name of the file into which to save the resulting figure. The *single_value_visualization* method assumes that the individual data-stores were populated during the analysis with *SingleValue* ADSs (which, as the name indicates, hold a single scalar value). It will automatically collate the data and show the value held in the *SingleValue* ADSs as a function of the varied parameters. If multiple *SingleValue* ADSs with different *value_name* parameters were produced during the analysis, it will automatically create multiple subplots, one for each of the encountered values of the parameter *value_name*. If the analysis script for the model demonstrated in this section contained analyses that created *SingleValue* ADSs with *value_names* parameter set to “*Mean(firing rate)*” and “*Mean(CV of ISI squared)*”, the above command would produce a figure such as the one shown in Figure 6.

Overall these examples demonstrate how seamlessly different components of Mozaik integrate, and the level of automation that is achievable thanks to this integration. We would like to emphasize that the extreme flexibility and broadness of the commands demonstrated in this sub-section critically depends on three of the defining features of Mozaik: the parameterizations system, the ADS system, and the rich metadata with which the various data structures are annotated throughout the Mozaik workflow.

## 5. Discussion

In this article we have presented Mozaik, a Python-based integrated workflow framework for spiking neuronal simulations. Much of the effort in detailed modeling projects in computational neuroscience is in managing the workflow and moving data and metadata between different simulation, analysis and visualization tools. Mozaik eliminates much of this effort by providing a framework that takes care of most of these low-level details, leaving the modeler to focus on the high-level concepts of models and experiments.

Mozaik leverages several broadly used neuroscience Python tools, thus increasing interoperability between current and future tools as well as increasing the possibility of replacing in future some of the Mozaik components with dedicated tools. Long-term, conditional on the continued convergence of neuroscience tools around common standards, the goal is for Mozaik to serve only as a glue between existing dedicated tools.

Mozaik is being used in real life modeling projects, and its scalability has been tested in a distributed HPC environment, both with large-scale network models (>10^5^ neurons and >10^8^ synapses) and large parameter searches (10^6^ independent simulation instances).

Since Mozaik uses PyNN to access simulator engines, the underlying speed of a Mozaik simulation run will inevitably depend on the performance of PyNN and, more importantly, the simulator backend selected for the given simulation. However, naturally, with the extra functionality Mozaik provides, it introduces extra overhead. To verify that this overhead is acceptably small, we have performed several test runs using the NEST simulator (currently the state of the art in large-scale point neuron simulation). As a benchmark we have selected the Vogels and Abbott (2005) model, an implementation of which is shipped with PyNN. This model does not require any sensory stimulation, which allows for a fair comparison between the pure PyNN and the Mozaik implementations, as sensory stimulation, which is handled by Mozaik, would in the case of a pure PyNN simulation have to be implemented by the user, thus not providing a well defined comparison reference. We ran both the Mozaik and pure PyNN simulations ten times with two different population sizes and the same number of synapses per neuron (10,000 and 100,000 neurons and ~200 synapses per neuron). For the smaller population size the mean run time of the pure PyNN implementation was 64.6 s (SD = 2.3) vs. 72.8 s (SD = 0.7) for the Mozaik implementation, indicating an overhead of about 13%. However, for the simulation of large populations the mean run time of the pure PyNN implementation was 1106.1 s (SD = 11.3) vs. 1123.5 s (SD = 5.8) for the Mozaik implementation, meaning that the overhead was now reduced to only 1%. Given that the Mozaik project is intended for large-scale simulations, we conclude that the overhead introduced is acceptable.

During the implementation of Mozaik it became clear that the most critical aspect of integrating the different aspect of the neuronal simulation workflow is the handling of metadata. In majority of Mozaik's components, notably in the analysis, visualization, and experimental protocol modules, the rich annotation of data-structures with metadata that flow between the components was instrumental in achieving the level of automation and flexibility demonstrated here. Unfortunately, there does not currently exist a common standard for interpretation of metadata in neuroscience (and more specifically in computational neuroscience). This lack of standard limits the ease of integration of current neuroscience tools, including Mozaik, as even though tools are able to exchange raw data thanks to common data formats such as those provided by the Neo package, the interpretation of metadata has to still be reconciled on an individual basis. It is the belief of the authors that creation of a common standard capturing the semantics of metadata is the next key technological advance that is required in the neuroscience community to achieve sufficient integration between the various tools.

As is clear from its architecture, Mozaik is a package that will be continuously developed and extended, and great care was put into its design to allow for ease of such incremental development. Specifically, the typical user will probably develop their own model components, stimuli, experimental protocols, analysis and visualization methods. We hope that users will contribute these additions back to the core Mozaik project, which will thus be able to provide a growing library of such resources. Furthermore, even the core API is likely to be changed in future as some parts of Mozaik will be taken over by dedicated tools, or forked out into independent packages. An example of such planned future changes is the replacement of the Mozaik datastore with dedicated database software, or linking of the Mozaik stimulus interface to one of the existing Python-based stimulation packages used in biological experiments. Finally, one of our short-term plans is the addition of a dedicated GUI for the analysis and visualization steps of the workflow and/or integration of Mozaik with one of the analysis and visualization libraries that are taking shape in the field.

## Funding

This work was supported by European Union projects FP7-269921 (BrainScaleS) and FP6-015879 (FACETS).

### Conflict of interest statement

The authors declare that the research was conducted in the absence of any commercial or financial relationships that could be construed as a potential conflict of interest.
